# Prevention and treatment of radiotherapy‐induced side effects

**DOI:** 10.1002/1878-0261.12750

**Published:** 2020-06-24

**Authors:** Lara Barazzuol, Rob P. Coppes, Peter van Luijk

**Affiliations:** ^1^ Department of Biomedical Sciences of Cells and Systems University Medical Center Groningen University of Groningen Groningen The Netherlands; ^2^ Department of Radiation Oncology University Medical Center Groningen University of Groningen Groningen The Netherlands

**Keywords:** brain, cardiopulmonary system, dose distribution, normal tissue effects, salivary gland

## Abstract

Radiotherapy remains a mainstay of cancer treatment, being used in roughly 50% of patients. The precision with which the radiation dose can be delivered is rapidly improving. This precision allows the more accurate targeting of radiation dose to the tumor and reduces the amount of surrounding normal tissue exposed. Although this often reduces the unwanted side effects of radiotherapy, we still need to further improve patients’ quality of life and to escalate radiation doses to tumors when necessary. High‐precision radiotherapy forces one to choose which organ or functional organ substructures should be spared. To be able to make such choices, we urgently need to better understand the molecular and physiological mechanisms of normal tissue responses to radiotherapy. Currently, oversimplified approaches using constraints on mean doses, and irradiated volumes of normal tissues are used to plan treatments with minimized risk of radiation side effects. In this review, we discuss the responses of three different normal tissues to radiotherapy: the salivary glands, cardiopulmonary system, and brain. We show that although they may share very similar local cellular processes, they respond very differently through organ‐specific, nonlocal mechanisms. We also discuss how a better knowledge of these mechanisms can be used to treat or to prevent the effects of radiotherapy on normal tissue and to optimize radiotherapy delivery.

## Graphical abstract

Here, we discuss the complexity of radiotherapy‐induced side effects and approaches for prevention or treatment. Radiation damage develops at a cellular level, progresses to intercellular interactions resulting in intra‐ and interorgan functional responses. These need different approaches for prevention or treatment, for instance intracellular signaling modification, sparing of specific stem cell‐containing areas or contributing organs, and stem cell therapies.

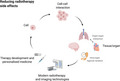

**Abbreviations**
ACE: angiotensin‐converting enzyme
ATM: ataxia telangiectasia mutated
BBB: blood–brain barrier ; CSF1R, colony‐stimulating factor‐1 receptor ;
CREB: cAMP response element‐binding
ECM: extracellular matrix
ESC: embryonic stem cells
FDA: food and drug administration ;
FGF: fibroblast growth factor
GDNF: glial cell line‐derived neurotrophic factor
HNC: head and neck cancer
IMRT: intensity‐modulated radiotherapy
MSC: mesenchymal stem cells
NOS3: nitric oxide synthase 3
OPC: oligodendrocyte progenitor cells
PSC: pluripotent stem cells
RAAS: renin–angiotensin–aldosterone system
ROS/RNS: reactive oxygen and nitrogen species
SASP: senescence‐associated secretory phenotype
SGZ: subgranular zone
SSPC: salivary gland stem/progenitor cells
SVZ: subventricular zone
TNF: tumor necrosis factor
VEGF: vascular endothelial growth factor
VMAT: volumetric modulated arc therapy

## 1 Introduction

The number of new cancer cases per year is estimated to rise to 22.2 million by the year 2030 worldwide [1], and about 12 million patients will receive radiotherapy as part of their treatment [2,3]. Although radiotherapy is well tolerated by most patients, some experience radiation‐induced side effects, the severity and frequency of which can be reduced by modern, more precise therapies, such as particle therapy and advanced image‐guided technologies. This improved precision can be used to minimize the radiation dose to normal tissue thereby reducing side effects, but can also be used for escalation of dose to poorly responding tumors without increasing the risk of side effects.

Currently, oversimplified approaches using constraints on mean doses and irradiated volumes of normal tissues receiving a specified dose are used to plan treatments with minimized risk of radiation side effects. Initially, consensus publications such as the Emami paper were the main sources for constraints [4]. More recently, these have been updated by systematic literature reviews, such as the one performed in the Quantec effort [5].

Modern radiotherapy techniques, such as intensity‐modulated radiotherapy (IMRT) and volumetric modulated arc therapy (VMAT) (Box 1), reduce the amount of normal tissue that receives a high dose of radiation but at the cost of large volumes of tissue receiving a low dose. In contrast, particle therapy (Box 1) allows to concentrate a high dose of radiation to the tumor while limiting the integral dose to normal tissues. As such, modern radiotherapy technologies offer greater precision but their optimal use requires radiation oncologists to have a better understanding of how these therapies affect normal tissue.

BOX 1 **Radiotherapy techniques**

From the 90s until today, radiotherapy has undergone a strong technological development aimed at improving precision of radiation dose delivery to the tumor while minimizing the dose to the normal tissue. By the end of the 90s, 3‐dimensional conformal radiotherapy (3DCRT) was introduced. In this technique, 3D imaging data prior to treatment are used to design a limited number of radiation beams with a fixed shape and uniform dose distribution matching the shape of the tumor volume. During the first decade of this century, this technique was enhanced into IMRT, which allows variations of dose within each beam, thus providing a new dimension of optimization. Typically IMRT also uses more beams than 3DCRT allowing a more conformal dose distribution. In the past decade, this was further developed into techniques irradiating while rotating the irradiator around the patient in VMAT. This technique results in arcs rather than discrete beams. Though technically all of these modalities use anatomical and more recently also functional information obtained before the start of treatment, utility of imaging obtained during the treatment was recognized. The use of such imaging is termed image‐guided radiotherapy. In parallel to the development of these photon‐based irradiation techniques, particle therapy, which is based on the use of ions, such as protons in proton therapy or carbon ions, can offer opportunities to further reduce the radiation dose to the normal tissue. In contrast to photons, particle therapy aims to achieve radiation dose deposition concentrated predominantly at a precise depth by exploiting the intrinsic physical properties of ions. This allows additional sparing of the normal tissue.

The risk of side effects on normal tissue depends on the radiation dose and the volume of normal tissue irradiated [6]. Recently, it has been shown that volume effects can be region‐dependent [7] and can even involve interactions between different organs [8,9]. Recent technologies might, for the first time, allow treatments to be optimized by taking into account such intra‐organ variations in sensitivity and interorgan interactions. In this review, we show that such optimization requires knowledge of the tissue and organ‐level mechanisms that are responsible for such regional variations and organ interactions. To this end, we address different aspects of the mechanisms of radiation‐induced normal tissue effects in general and more specifically of the salivary glands, cardiopulmonary system, and brain. These three organs exhibit similar local cellular responses but nevertheless differ strongly in their response to radiotherapy due to fundamental differences in tissue organization and in the consequences of tissue damage for their function. We discuss these consequences, focusing on their implications for the prevention and treatment of radiation‐induced side effects.

## 2 Radiation‐induced side effects

Radiation activates a damage repair cascade in normal tissues. This cascade initiates with the DNA damage response that includes apoptosis, mitotic cell death, and cellular senescence [10], and is followed by a perpetual cytokine cascade, which induces inflammation and excessive extracellular matrix (ECM) and collagen deposition, processes that are largely modulated by reactive oxygen and nitrogen species (ROS/RNS) imbalance and tissue hypoxia [11]. The side effects of radiotherapy in normal tissue can be divided into early (or acute) and late responses, depending mostly on tissue turnover time and their modulation by processes that mimic a wound healing response [11]. Early (or acute) side effects occur during, immediately after, or soon after (within weeks of) radiotherapy treatment [11,12]. Early side effects are often reversible when the dose is limited and tissue turnover is high, such as in the oral mucosa [13] and gut, or partly reversible, such as in lungs (pneumonitis) [14], skin [15], and brain (memory loss and fatigue [16]).

Late normal tissue side effects are defined by their occurrence several months to years after radiotherapy [11,12]. Late side effects are in general chronic and often progressive, leading to a reduction in patients’ quality of life following treatment. These are, therefore, often employed to determine radiation dose limits [11]. In contrast to early side effects, the time to response of late‐responding tissues depends on the dose and is modulated by processes such as cellular senescence, chronic inflammation, hypoxia, and fibrosis [11]. All of these responses subsequently inhibit the regenerative potential of the tissue. Importantly, fibrosis is involved in the pathogenesis of side effects in most tissues, such as heart [17], lung [14], and liver [18].

Although different normal tissues may share very similar localized cellular processes, they may respond very differently owing to organ‐specific, nonlocal mechanisms, such as loss of peripheral tissue secondary to loss of stem cells located in specific regions and functional dependence between organs. In the following sections, we describe such responses for three different tissues, the salivary gland, lung, and brain. We also illustrate how these responses can offer unique opportunities for therapeutic and preventative strategies.

## 3 Salivary glands

Most head and neck cancer (HNC) patients are treated with radiotherapy alone, or in combination with chemotherapy and/or surgery. This often results in the unavoidable co‐irradiation of the peripherally positioned salivary glands. Forty percent of HNC patients receiving IMRT will experience moderate or severe xerostomia (‘dry mouth syndrome’), resulting from hyposalivation, leading to alterations in speech and taste, difficulties with mastication and deglutition, and an increased risk of developing oral infections and dental caries [19, 20, 21]. These sequelae severely hamper the quality of life of affected patients.

### 3.1 Cellular and tissue responses over time

**Fig. 1:**
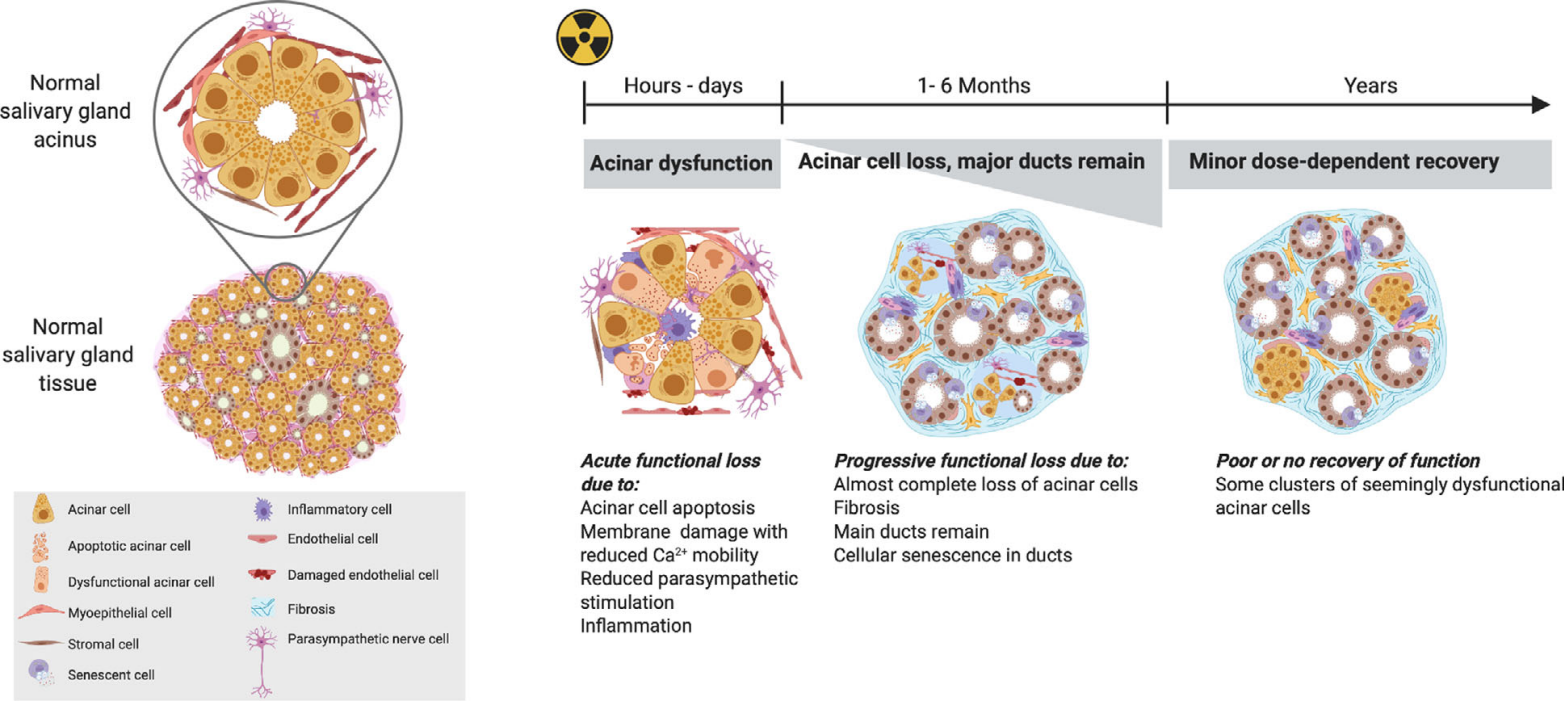
A schematic of the cellular and tissue responses of the salivary gland to radiotherapy over time. (Left panel) The left panel depicts a model of a salivary gland and shows the structure of the acinus, which when enlarged features the different cell types it is composed of. (Right panel) The early and late responses of salivary gland tissue to radiotherapy. The early response (which occurs within hours or days) is completely different mechanistically to the late response. The early response is too rapid to be explained by mitotic failure or related cell death and seems to be due to failure of vasculature function, parasympathetic nerve function, acinar cell signal transduction, and possibly inflammation and acinar cell apoptosis (which is limited, depending on the experimental model used). The later effects (which occur > 30 days after radiotherapy) result from acinar cell loss, which coincides with chronic inflammation and fibrosis. Depending on the radiotherapy dose used, some morphological recovery might follow, as shown by the appearance of acinar cell clusters.

Salivary glands contain saliva‐producing mucous and serous acinar cells, myoepithelial cells, duct cells, cholinergic and adrenergic nerve fibers, blood vessels, and supporting stromal tissue [22,23], which can all be affected by irradiation. Interestingly, although salivary gland parenchymal cells are mostly postmitotic with a cell turnover time of 60–120 days, their response to radiation is acute as observed both in rodents [24] and in humans [25] and is followed by a later response, which is induced by different mechanisms [26,27] (Fig. 1). The early response cannot be due to mitotic failure and has been attributed to several abnormalities in murine and rhesus monkey: the apoptosis of acinar cells [28,29], the membrane damage‐induced dysfunction of acinar cells [24,26], the impairment of microvessels [30], and reduced parasympathetic signaling [31]. A major characteristic of late radiation‐damaged salivary glands is the accumulation of chronic inflammation and fibrosis and consequent tissue dysfunction and atrophy [11,24] (Fig. 1). This coincides with a lack of regenerative potential of salivary gland stem/progenitor cells (SSPCs) [32]. Indeed, the radiation‐surviving SSPCs in and outside of the radiation field have been shown to determine the regenerative capacity of the gland post‐treatment [32,33]. Interestingly, senescent cells accumulate in the murine salivary gland ducts [34], where SSPCs seem to reside [32]. These senescent cells develop a unique secretory phenotype, called senescence‐associated secretory phenotype (SASP). The SASP includes several proinflammatory and profibrotic growth factors [10] and is associated with reduced tissue regenerative capacities, inflammatory processes, and fibrosis [35]. In rodent models, acini have been shown to have some regeneration capacity even 1 year after treatment, as large acinar cell clusters have been found in irradiated salivary gland tissues (Fig. 1) [36,34]. This is probably due to acinar cell proliferation. However, the resulting clusters do not seem to be functional [36]. While there is relative consensus in the field regarding the late effects of radiation on salivary glands, there is less clarity about the early effects, which depend on the preclinical model used. The salivary glands of different rodent species and strains respond quite differently to irradiation. FVB mice [38] and Wistar rats [26, 27, 28] have a clear early response [38], whereas C57BL/6 mice have a relatively mild early response but a strong late response [39]. Additionally, different radiation dose tolerance and responses depend on whether the radiation field is localized or includes the whole head [40].

### 3.2 Therapeutic approaches

Our increased level of understanding of radiation‐induced damage has led to a multitude of therapeutic strategies to ameliorate salivary gland radiation damage. These have recently been reviewed by Jensen *et al*. [41], here, we focus on some that are related to the above‐described mechanisms.

Many radical scavengers have been tested in a variety of models. ROS/RNS scavengers aim to reduce the effective radiation dose to the tissue, thereby potentially sparing normal tissue cells but not the tumor. One such scavenger is amifostine, which in rat salivary glands shows amelioration of function loss depending on the irradiated region in rat salivary glands [42] and improved protection when it is injected in a retrograde manner in the secretory ducts [43], where SSPCs seem to be located [32]. However, amifostine clinical trials have been inconclusive, owing to limited statistical power or a lack of proper control arms [41]. In addition, amifostine has serious side effects at the moment of administration, such as nausea, and probably cannot be administered in the effective dose range used in animal experiments [44]. An alternative with less side effects could be tempol, which has been shown to protect the murine salivary gland and not the tumor [45]. Other strategies have been developed to optimize the salivary gland’s regenerative potential after radiotherapy. Most of these treatments stimulate the proliferation of the remaining SSPCs, limiting their use to (parts of) the tissue that have received a relatively low dose [46]. These approaches encompass both pharmacological agents that stimulate the parasympathetic response of the gland, such as pilocarpine, and growth factors that stimulate proliferation [46]. The saliva secretion inducing sialagogue, Pilocarpine, has been relatively well studied, both as a treatment before and after radiotherapy. Animal and human studies show that this drug produces some improvement in salivary flow, which seems again to be limited by the maximum dose received [47,48] and which probably relies on improving the proliferation of the remaining radiation‐surviving cells [49]. Similarly, growth factors, such as insulin growth factor 1 [50], keratinocyte growth factor [51], as well as cytokine producing mesenchymal stem cells (MSCs), have been shown to improve salivary gland function after relatively low‐dose irradiation by stimulating the proliferation of radiation‐surviving cells. MSCs derived from different sources, such as bone marrow or adipose tissue, have been associated with the regeneration of radiation‐damaged normal tissues [52] including salivary glands [53,54]. A recent phase I/II clinical trial has shown the clinical feasibility and a marginal effect of such a therapy in the treatment of postradiotherapy hyposalivation [55]. However, since MSCs do not transdifferentiate into salivary gland cells but rather stimulate remaining surviving cells to proliferate, their action depends on the number of surviving SSPCs and their effect might be limited to the lower radiation dose regions [46]. Interestingly, MSCs have been shown to remodel radiation‐induced fibrosis [52]. Therefore, sequential or combined treatment with senolytics, drugs that kill senescent cells, and MSCs might improve the radiation‐damaged salivary gland environment for transplantation. The dependence of such therapies on the viability of remaining stem cells demands new research to enhance the number of surviving SSPCs.

Stem cell‐based therapy may provide a means to reduce radiation‐induced hyposalivation in patients after radiotherapy treatment [32]. Recently, we have shown the potential of stem cell therapy to ameliorate radiation‐induced hyposalivation in mice using expanded murine and human autologous adult SSPCs [56, 57, 58]. Interestingly, the positive effect of human SSPCs was partially due to the remodeling of the tissue [58]. SSPCs, however, cannot be obtained from patients with late radiation toxicity. A solution for this would be to use episomal reprogrammed somatic cells, such as blood mononuclear cells [59], that can generate pluripotent stem cells (PSCs), which are able to differentiate into every cell type in the body [60]. Similarly, embryonic stem cells (ESCs) have recently been shown to be able to differentiate into salivary gland cells [61], opening up novel avenues for regenerative medicine. A stem cell‐based approach to treating the side effects of radiotherapy on normal glands might be most effective when combined with the remodeling of the radiation‐damaged salivary gland environment.

### 3.3 Preventing radiation‐induced damage of salivary glands

Knowing where SSPCs are localized could help to prevent radiation‐induced salivary gland dysfunction. We have shown that the stem cells of the rodent and human parotid salivary gland localize to a specific region where the main excretory ducts are [7]. Sparing this region from radiation during radiotherapy for HNC is currently being evaluated in a double‐blind randomized clinical trial [62], with promising preliminary results. However, the complete sparing of the area that contains the highest proportion of SSPCs proved to be difficult due to the close vicinity of the tumor. This and the finding that these stem/progenitor cells might be very sensitive to low doses of irradiation [63] warrant the use of very accurate radiotherapy technologies, such as proton therapy (Box 1). The above‐described approaches have all been developed after obtaining a deeper knowledge of the response of the salivary gland to irradiation. Expanding this knowledge might, in the future, result in patient‐specific approaches that can prevent or ameliorate radiotherapy‐induced hyposalivation.

## 4 Cardiopulmonary system

**Fig. 2:**
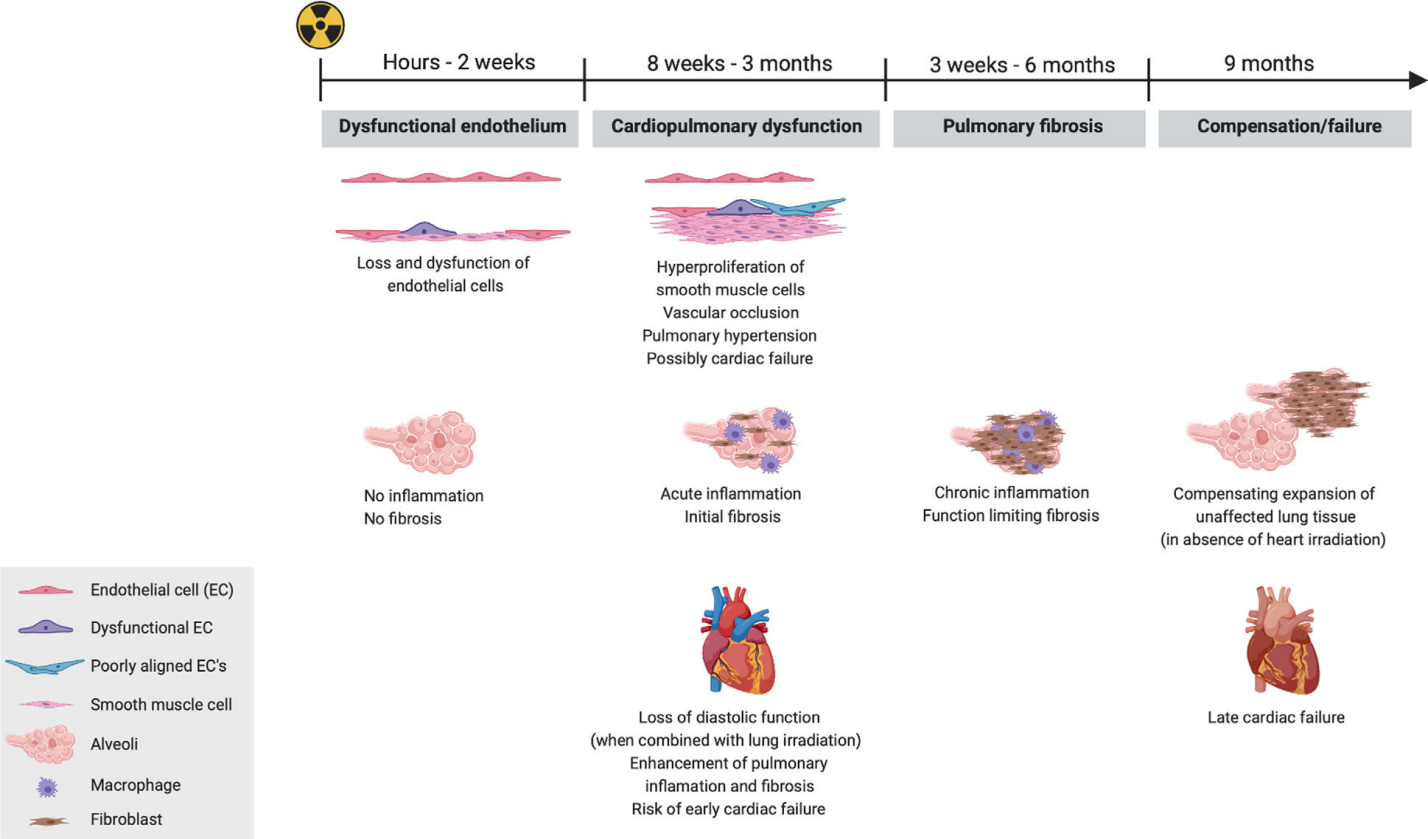
A schematic of the side effects of radiotherapy on the cardiopulmonary system over time. Shown are the cellular, tissue, and organ responses over time in the tissues of the lung and heart. The loss and dysfunction of lung vascular ECs are the first visible forms of damage in the irradiated lung. This is followed by acute inflammation and, depending on the dose, the first signs of fibrosis. These local processes are aggravated by loss of diastolic function if the heart is irradiated concomitantly. In the subsequent months, lung damage progresses and features chronic inflammation and function‐limiting fibrosis. The resulting dyspnea might resolve at later time points by compensatory inflation of nonirradiated parts of the lung. The adequacy of this compensatory response, however, depends on the irradiated lung volume. In addition, cardiac irradiation might also lead to later onset cardiac failure.

Treatment of thoracic cancers with radiotherapy can cause side effects. Traditionally, early pulmonary and late cardiac damage has received the most attention [64]. The clinical sequelae of radiation lung injury usually start with the acute onset of radiation pneumonitis at 2–6 months after radiotherapy with symptoms that range from cough, fever, and dyspnea to even death from respiratory failure. Radiation‐induced lung fibrosis often develops subclinically from several months to years after radiotherapy. In rodents, symptoms of toxicity manifest as increased breathing frequency [65, 66, 67]. Several inflammatory responses initiated by radiation and radiation‐induced damage contribute to radiation pneumonitis [68,69]. Acute alveolar and interstitial inflammation leads to the loss of type I epithelial cells and endothelial cells, while inducing the proliferation of type II epithelial cells. These events initiate a cascade of inflammatory cytokines, which plays an important role in radiation pneumonitis [70]. This can be aggravated by combined treatment with chemotherapeutic agents [70]. Furthermore, beginning at 4 weeks after irradiation, an increase in ECM collagen deposition can be observed in the lungs of mice [71] (Fig. 2, Box 2).

BOX 1 **Glossary**

*Pneumonitis*: inflammation of lung tissue.

*Radiation pneumonitis*: pneumonitis caused by radiation.

*Episomal reprogramming*: system that reprograms somatic cells into induced PSCs, which are able to differentiate into almost any type of cell in the body.

The risk and severity of lung radiation side effects depend on the dose and volume of the tissue involved [65,72, 73, 74]. Therefore, technical advances that enable treatment optimization are currently used to reduce dose and volume of normal tissue receiving radiation.

### 4.1 Cellular and tissue responses

In the 1990s, Travis *et al*. [75] hypothesized and confirmed that the consequences of irradiating the lung might vary depending on which part of the lung was irradiated. Their studies in mice found that responses to the irradiation of basal subvolumes of the lung were consistently more severe than those observed after the irradiation of apical subvolumes. Based on these results, they hypothesized that this variation originated from a nonuniform distribution of alveolar tissue over the lung [76]. The latter hypothesis was indeed confirmed in rat studies, in which irradiation of the lateral parts of the lungs resulted in more severe respiratory dysfunction than did irradiation of the medially located parts of the lung that also contain the major airways [66]. However, interestingly in the same model, it was also observed that part of the regional variation in response could be explained by variations in dose in the heart [9,77]. Although also in patients a regional variation in the risk of radiation pneumonitis was observed, the data cannot distinguish between a role of alveolar distribution and involvement of the heart [78].

Since the heart and lungs are linked by the cardiopulmonary circulation, it was hypothesized that damage in these two organs after radiotherapy is mediated by changes in the cardiopulmonary circulation. Indeed, in rats it was found that heart irradiation reduced left ventricle diastolic function, leading to signs of congestion in the lungs that closely resembled the classical signs of radiation pneumonitis, such as shortness of breath, inflammation, and fibrosis [8]. Interestingly, also in rats it was shown that lung irradiation damages the endothelial cell (EC) layer of the pulmonary microvasculature within the first 2 weeks after irradiation, which precedes parenchymal damage [79]. These damages occur both in irradiated and spared lung tissue [79]. It was suggested that irradiation induces EC loss and that this leads to a contraction of the vasculature, which subsequentially results in an increase in pulmonary pressure in the unirradiated part of the lung. This increased pressure induces the sheering of ECs, much like that observed in pulmonary hypertension models [80,81]. EC injury might initiate or mediate structural changes in pulmonary vasculature, as described for pulmonary hypertension [82,83]. Indeed, pronounced vascular remodeling, including muscularization, adventitia thickening, and neointima formation throughout the lungs, was observed to result in increased pulmonary vascular resistance, leading to pulmonary hypertension [8,79]. The pathological features of the pulmonary vasculature were highly specific for pulmonary arterial hypertension. Pulmonary arterial hypertension can also impair left ventricle function, further aggravating the symptoms of cardiopulmonary dysfunction [8]. These effects depend on the irradiated lung volume, possibly pointing to a critical role for the amount of irradiated vasculature in the etiology of cardiopulmonary dysfunction [79]. As a consequence, limiting the irradiated volume may be more effective in preventing cardiopulmonary dysfunction than reducing the radiation dose within irradiated volumes.

Interestingly, cardiac irradiation has also been shown in a rat model to cause early interstitial and perivascular fibrosis of the heart in combination with loss of diastolic function [8]. Loss of diastolic function, and its associated congestion in the pulmonary circulation, increases the risk of cardiopulmonary dysfunction. When combined with pulmonary hypertension, loss of diastolic function increases the risk of cardiac failure [8].

In the ongoing CLARIFY study, the occurrence and impact of these and potentially other disturbances of cardiopulmonary physiology on patients are currently studied in a large prospective cohort study in lung and esophageal cancer patients using pre‐ and post‐treatment echocardiography, cardiac magnetic resonance imaging, and blood biomarkers [84].

These findings indicate that in the development of radiation damage, the heart and lung must be considered as an integrated system. This broader view of integrated responses of organs and organ systems provides us with novel opportunities to optimize radiotherapy treatment, as well as treatment of toxicity. Firstly, the functional interaction that exists between the heart and lung calls for the combined dose distribution in both organs to be optimized for the underlying systemic changes. Secondly, the process underlying the interaction between heart and lung provides novel targets for interventions to prevent toxicity.

### 4.2 Therapeutic approaches

Multiple approaches have been explored to reduce pulmonary toxicity but very few have been used in the clinic. Nevertheless, the mechanisms described above could inform the development of novel preventive or treatment measures.

For the nononcological patient, the prolonged activation of the renin–angiotensin–aldosterone system (RAAS) plays an important role in the progression of cardiac failure [85]. Consequently, inhibiting various components of the RAAS is a cornerstone of current treatments for heart failure [85,86]. The inhibition of angiotensin‐converting enzyme (ACE) to prevent the conversion of angiotensin I into angiotensin II and the activation of downstream mechanisms has been the most common approach used to achieve this.

Cardiac irradiation causes loss of diastolic function with detrimental consequences for the lung, as observed in a rat model [8,87]. Given this, could ACE inhibition be a promising strategy by which to reduce or prevent the failure of the cardiopulmonary system after thoracic radiotherapy? This does indeed appear to be the case. In a rat model, the ACE inhibitor, captopril, was observed to reduce dyspnea after whole‐thorax irradiation [88,89]. In experiments where radiation doses in heart and lung were varied in a controlled manner, this effect was shown to be likely achieved by reducing interstitial and perivascular fibrosis in the heart, leading to the preservation of diastolic function [87]. These results suggest that the RAAS is indeed involved in the development of radiation‐induced loss of diastolic function. Moreover, this finding suggests that treatments for conditions that lead to cardiac failure in nononcological patients might be potentially useful for preventing radiotherapy‐induced cardiopulmonary complications.

### 4.3 Preventing radiation‐induced damage in the cardiopulmonary system

As indicated, research on the regional responses of the lung was inspired by the idea that such regional responses might offer opportunities to reduce, or even prevent, normal tissue toxicity by avoiding the most important regions of the lung [75]. Indeed, this idea led to clinical studies in which risk estimation improved, the use of lung doses was replaced by a hybrid model that incorporated the local function of the lung and that optimized treatment strategies by avoiding vital lung tissue. This was achieved by moving the radiation dose to parts of the lung with a lower contribution to function [90].

However, as described in the previous section, recent animal work illustrates that cardiac and pulmonary toxicity can no longer be seen as separate entities and that both organs need to be spared. Unfortunately, the ability of photon‐based treatment technology to achieve this is limited due to dose deposited beyond the tumor volume. Interestingly, the peaking of the dose distribution of particles near the end of their penetration depth and the lack of dose beyond is expected to offer unprecedented opportunities to achieve this [91,92].

## 5 Brain

**Fig. 3:**
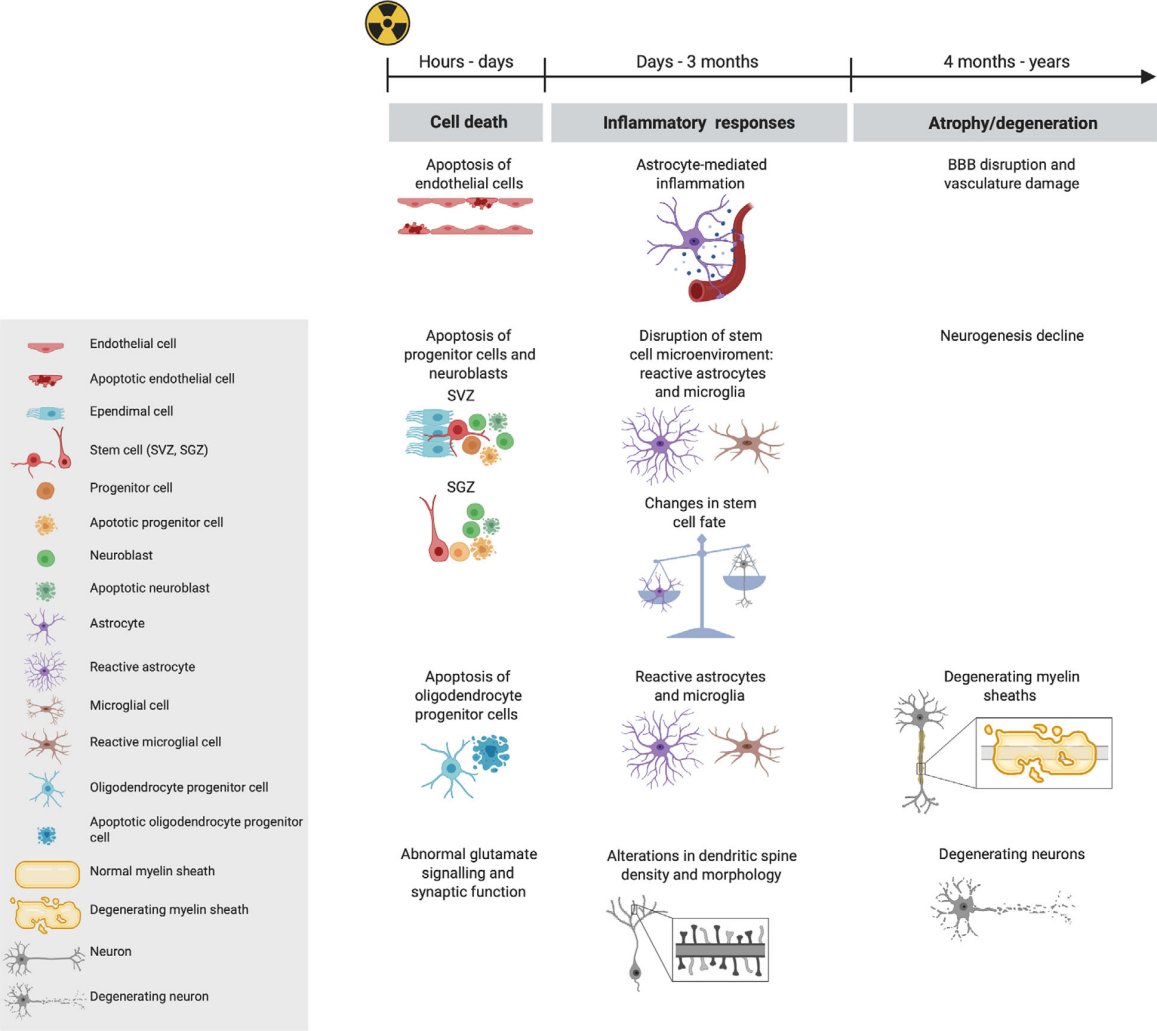
A schematic of the cellular and tissue responses of the brain to radiotherapy over time. Radiation causes multiple effects in the brain, including vascular damage, neurogenesis decline, white matter damage, and neuronal damage. Within hours after irradiation, cell death, largely via apoptosis, occurs in ECs, progenitor cells, and neuroblasts of the SVZ and SGZ, and in OPCs. Neurons exhibit abnormal glutamate signaling and synaptic function relatively early after irradiation, alongside alterations in dendritic spines and morphology. These early responses are followed by an inflammatory response that is characterized by the release of cytokines, and the reactivity of astrocytes and microglial cells. This inflammatory response can contribute to both early and late effects that affect different cell types and their interactions.

Radiotherapy‐induced neurocognitive dysfunction represents the major side effect of cranial radiotherapy in adult and pediatric cancer survivors, affecting school performance, employment, and independent living [93]. The brain is mostly formed by postmitotic neurons and glial cells. Glial cells primarily consist of the following cell types: astrocytes, which support neuronal function;oligodendrocytes, which are responsible for coating axons with myelin; and microglia, which are the resident macrophages of the brain. The brain also harbors a limited number of neural stem and progenitor cells in two restricted regions of adult neurogenesis. Importantly, the brain is isolated from the rest of the body's bloodstream by the blood–brain barrier (BBB), which is formed of highly selective junctions between ECs. Although it is difficult to dissect the contribution of each cell type and their interaction to the pathogenesis of the neurocognitive dysfunction that occurs as a late response (> 4 months) after radiotherapy, these cell types have been shown to be all somehow affected by radiation (Fig. 3).

### 5.1 Cellular and tissue responses over time

Endothelial cells form the inner layer of blood vessel walls and, together with mural cells and astrocytes, ensure cerebral blood flow and BBB integrity [94]. Within hours after irradiation, in rodent models, ECs apoptose in a p53‐independent manner via the acid sphingomyelinase pathway, leading to the early disruption of BBB permeability [95,96]. This is followed, at 1–3 months after irradiation, by the irreversible disruption of the BBB, which is linked to persistent inflammation modulated by tumor necrosis factor (TNF)‐alpha and vascular endothelial growth factor (VEGF) overexpression in the astrocytes surrounding the ECs [97]. This delayed response is often restricted to white matter regions and precedes the development of radiation‐induced necrosis.

As in the salivary gland, the adult brain contains slowly proliferating stem and progenitor cells. Neurogenesis in the adult mammalian brain primarily occurs in two regions, the subventricular zone (SVZ) adjacent to the lateral ventricles and the subgranular zone (SGZ) in the dentate gyrus of the hippocampus. Within hours after irradiation, ataxia‐telangiectasia mutated (ATM) and p53‐dependent apoptosis of neural progenitor cells is observed in the SVZ [98,99] and in the SGZ region of rodent models [100]. In the longer term (> 2 months postirradiation), a change in cell fate differentiation from a neuronal to a glial fate is also observed [100]. This delayed response seems to be due to changes in the stem cell niche microenvironment, which include elevated levels of activated microglia and a disrupted vasculature that affects the regenerative potential of the remaining stem cells [100].

Glial cells include oligodendrocytes, astrocytes, and microglia. Oligodendrocyte progenitor cells (OPCs) in rodent models, similar to the progenitor cells of the SVZ and SGZ regions, have been shown to apoptose hours after irradiation. This in the long‐term (> 4 months postirradiation) results in the irreversible depletion of mature myelinating oligodendrocytes, leading to white matter damage [101,102]. This effect seems to be partially mediated by the upregulation of VEGF by reactive astrocytes in proximity to white matter regions [103]. Astrocytes and microglia in rodent models have indeed been shown to similarly respond to radiation with an initial (within hours) overexpression of TNF‐alpha and other cytokines, leading over time to chronic neuroinflammation characterized by reactive astrogliosis and subsequent microglial activation lasting for years after irradiation [104,105].

Despite being the largest cell population in the brain, a limited number of studies have focused on the direct impact of radiation on postmitotic neurons. Extensive changes in mouse neuronal dendritic and spine morphology have been reported after doses of 1 to 10 Gy, starting as early as 10 days after irradiation and progressively worsening thereafter [106]. Among the different types of dendritic spines, early filopodia membranous protrusions were most sensitive to radiation, thus possibly hindering their development into mature dendritic spines. These morphological changes were accompanied by a decrease in the presynaptic marker synaptophysin, followed by an upregulation of the synaptic protein PSD‐95. Abnormal glutamate signaling (as early as 1 h after irradiation with 10 Gy) has been proposed as being a contributing factor to radiation‐induced synaptic changes that lead to excitotoxicity by excessive synaptic stimulation [107]. However, how radiation directly impacts the dendritic spines remain to be fully elucidated and other intermediated responses are likely mediating this effect. Additionally, whether all of the above responses faithfully recapitulate what is occurring in the human brain after radiotherapy treatment still remains unanswered.

### 5.2 Therapeutic approaches

Many therapeutic strategies have been explored preclinically in the quest to ameliorate the radiotherapy‐induced neurocognitive sequelae. For example, in rats, the PPAR‐gamma agonist, pioglitazone, and the ACE inhibitor, ramipril, have been shown to indirectly improve neurocognitive dysfunction, although without directly improving the already damaged brain vasculature [108, 109, 110]. In patients with brain tumors, pioglitazone and ramipril have been, or are currently being, tested in phase I and II clinical trials [111] (ClinicalTrials.gov Identifier: NCT03475186).

With the goal of improving tissue regeneration, stem cell transplantation therapies have also been explored in the brain. Preclinical studies using either human embryonic or neural stem cells in rodent models have demonstrated the ability of these cells to integrate in the brain and to differentiate into neurons and glial cells, leading to improved neurocognitive function [112,113]. The underlying mechanisms of this benefit are not fully established and seem to not be limited to the replacement of the lost or damaged cells but rather to include the microvesicle‐mediated release of trophic factors (such as glial cell line‐derived neurotrophic factor, GDNF, and basic fibroblast growth factor, FGF) by the transplanted stem cells themselves [114]. Intranasally delivered human MSCs have also been used and have recently been shown to effectively improve neurocognitive function after irradiation in mice, conferring protection against several responses, including radiation‐induced persistent cAMP response element‐binding (CREB) activation, inflammation, oxidative stress, and neuronal loss [115].

Pharmacological interventions using food and drug administration‐approved psychiatric medications have also been explored as possible ways to reduce the long‐lasting impact that radiation has on adult neurogenesis. For example, in mice lithium [116] has been shown to reduce apoptosis of neural stem and progenitor cells in the SGZ, and in rodent models melatonin has been shown to increase the engraftment of neural stem cells in the SVZ [117,118]. Running‐based exercise has also been shown to improve neurogenesis in mice after irradiation [119]. The underlying mechanisms of how these pharmacological and physical interventions increase neurogenesis, however, remain to be fully elucidated and yet to be confirmed in patients.

A promising preclinical strategy to overcome radiation‐induced white matter damage is the transplantation of human embryonic stem cell‐derived OPCs into the corpus callosum and cerebellum of irradiated rats [120]. This was shown to lead to an improvement in neurocognitive and motor functions. However, the clinical translation of such an approach remains challenging. In mice, the depletion of microglia using PLX5622, a dietary inhibitor of colony‐stimulating factor‐1 receptor (CSF1R), was shown to prevent radiation‐induced neurocognitive dysfunction [121]. Other interventions to reduce glial cell reactivity have focused on reducing inflammation using nonsteroidal anti‐inflammatory drugs or selective inhibitors of proinflammatory cytokines.

In terms of neuronal damage, the administration of memantine, which blocks the glutamate receptor NMDAR, in mice just before irradiation prevented some of the radiation‐induced synaptic alterations [107]. Clinically memantine has been shown to improved neurocognitive function over time in patients receiving whole‐brain radiation therapy [122].

### 5.3 Preventing radiation‐induced damage in the brain

One strategy to prevent radiation‐induced side effects is to physically minimize the dose and volume of irradiated normal brain tissue or to minimize the exposure of specific brain structures to irradiation. This can be achieved using modern radiotherapy technologies, such as particle therapy. Our current knowledge of the potential role of different neuroanatomical structures in the pathogenesis of radiotherapy‐induced neurocognitive decline is largely limited to the hippocampus and its memory function [93]. However, other brain structures are likely to contribute to the complex neurocognitive sequelae that occur after radiotherapy, especially in pediatric patients, in whom a large proportion of tumors are located in the posterior fossa [123]. Future preclinical and clinical efforts should focus on discerning the contribution of different brain structures to radiation‐induced neurocognitive dysfunction in order to make optimal use of increasingly advanced radiotherapy technologies.

Another area that is little understood is the contribution of genetic variation to neurocognitive outcome. This topic has been recently reviewed in Ref. [124]. Strikingly, and possibly due to methodological issues, very little research has focused on trying to identify genes that are specifically associated with the development of radiotherapy‐induced neurocognitive dysfunction, with only one study showing a potential role for nitric oxide synthase 3 (NOS3) 894Thomozygosity [125]. Future research on the contribution of germline mutations, as well as the role of tumor genetic variation, is urgently needed to identify those patients who are most at risk of developing neurocognitive impairment and to offer them personalized preventive or therapeutic options.

## 6 Concluding remarks

In this review, we have discussed how recent developments in understanding how radiotherapy causes toxicity in normal tissue can be understood by describing three normal tissues at risk of such toxicity. The increasing availability of high‐precision radiotherapy is changing the way that we look at its effects on normal tissue. Changes to dose distribution and our increasing knowledge of the local and nonlocal effects of radiotherapy on normal tissue warrant the use of a different approach to prevent and/or to treat these effects. One of the commonalities of the three tissues described in this review is the occurrence of (micro‐) vascular effects. These effects have been described in many tissues but have received relatively little attention when compared to inflammatory processes and fibrosis. Since vascular effects might result in volume‐dependent within‐tissue and between‐tissue responses, the incorporation of such mechanisms in dose distribution planning would improve treatment outcomes. Such a strategy, however, requires quantitative information on the associated responses in patients that can only be obtained in translational clinical studies, such as our ongoing study into cardiopulmonary toxicity (acronym CLARIFY) [84]. Another effect, possibly one that is not independent of the vascular effect, is the stem cell region‐dependent regenerative potential of some normal tissues. Here also, altering dose distributions and sparing region that contain stem cells (as described in the salivary gland and brain) might also result in the further optimization of radiotherapy. Sparing or reducing the dose that somatic stem/progenitor cells are exposed to could result in the recovery of even late‐responding tissues. Their subsequent stimulation, using cytokines or MSCs, could further improve the regenerative potential of, and prevent excess damage to, normal tissue. In addition, regenerative therapies, based on the replacement of the damaged tissue‐specific stem/progenitor cells, might provide a means by which to further optimize the regenerative potential of the irradiated tissue. Recently, the development of *in vitro* tissue resembling models, such as organoids and organs‐on‐chip, derived from human tissue‐specific adult stem cells and from ESCs/PSCs and containing different cell types, including stem/progenitor cells and specialized differentiated functional cells [126], open up endless possibilities for modeling radiation‐induced side effects.

A deeper knowledge of the mechanisms that underlie normal tissue damage might also help to develop better preventive and therapeutic strategies. We need to progress from understanding local molecular/cellular events toward having a better understanding of tissue and organ interactions; this progress does not occur automatically and needs to be supported by subsequent translational research using animal models or tissue resembling models [63,127]. Typically, studies of the importance of different structures, in particular for the adult and pediatric brain, are needed to define (functional) structures that need to be spared or that can tolerate a somehow larger dose. Moreover, these structures are very likely to be interacting with each other, hence increasing the complexity of such studies. The most optimal animal models should also be used to address specific research questions. Although mice are more available and amenable to genetic manipulation, they might be too small to achieve accurate irradiation fields and are also characterized by significant differences in responses between strains. Rats or even larger experimental animals might be needed to design preclinical studies to test the optimal use of modern radiotherapy technologies. Genetic clinical studies to identify those patients that are most at risk of developing late side effects (although not reviewed here) are certainly of importance and, when possible, should be validated in combination with animal studies considering the above‐described principles.

The here‐described therapeutic and preventative strategies warrant further translation; however, many have yet to reach the clinic. The progress of an idea from the laboratory to the clinic and back to the laboratory to address further questions requires a well‐connected multidisciplinary team, which regretfully is often lacking within one institute. Alongside this, it seems that findings from well‐controlled experiments in animal models optimized for specific targets are very difficult to mimic in clinical studies that involve a diverse group of patients, who are often suffering from underlying diseases. Obtaining insight into the potential relevance of preclinical ideas using small clinical proof‐of‐concept studies, such as the MRI‐HART study [128], which precedes the much larger ongoing CLARIFY study [84], is essential for optimizing the design of clinical studies and for maximizing the probability that preclinical findings will reach clinical practice. However, such translational paths require long‐term commitments from both the laboratory and the clinic. Offering opportunities or work settings that allow a better understanding of each other’s fields, for instance, by spending internships in the laboratory or in the clinic, may help to achieve this.

Although the translation of preclinical research remains a challenge, several of the above‐described research discoveries are slowly entering the clinic. Stem cell‐sparing trials, such as the one described in Ref. [62], are currently being performed and some stem cell therapies are close to or in phase I/II clinical trials. However, the future improvement of combined biology and modern radiotherapy technologies depends on a constant, intense effort based on interdisciplinary and international collaborations between all the fields involved in (radiation) oncology.

## Conflict of interest

The authors declare no conflict of interest.

## Author contributions

LB, RPC, and PVL equally contributed to the writing of the manuscript and design of the figures.
